# S179. PROGNOSTIC UTILITY OF MULTIVARIATE MORPHOMETRY IN SCHIZOPHRENIA

**DOI:** 10.1093/schbul/sby018.966

**Published:** 2018-04-01

**Authors:** Mingli Li, Xiaojing Li, Deng Wei, Yinfei Li, Liansheng Zhao, Xiaohong Ma, Yingcheng Wang, Hua Yu, Ya-jing Meng, Qiang Wang, Lena Palaniyappan, Tao Li

**Affiliations:** 1 Mental Health Center, West China Hospital of Sichuan University; 2 University of Western Ontario

## Abstract

**Background:**

Groups of spatially distributed regions show shared variance in morphometric properties (e.g. grey matter volume) among subjects, thus forming independent morphometric ‘sources’ or covariance-based networks. Source based morphometry is a multivariate approach that is based on independent component analysis, and accounts for the inter-relationahsip among different brain regions while filtering out noisy artefactual effects of mass univariate voxel-based approaches. We have previously demonstrated that with multivariate SBM, it is possible to identify the structural basis of subtle psychopathological features such as formal thought disorder, whose anatomical correlates have been hitherto elusive. In the current study, we use multivariate SBM to identify the morphometric sources in drug-naïve first episode subjects that show progressive changes that predict symptom change over 1 year.

**Methods:**

63 first-episode, drug-naive patients with schizophrenia underwent brain magnetic resonance imaging scans at baseline (T0) and rescanned after 1 year follow-up (T1). Positive and Negative Syndrome Scale (PANSS) was used to assess their psychopathology. Source based morphometry (SBM) was performed to analyze the gray matter volume (GMV), paired T contrasts for loading coefficients of GMV were constructed to detect the components that showed a significant effect of time. The change in PANSS scores between baseline and 1 year was expressed as a ratio of the scores at baseline - adjusted change scores for positive symptoms (POS%), negative symptoms (NEG%) and disorganization symptoms (DISORG%), with each domain score derived using van der Gaag’s 5-factor approach. Multiple regression analysis was conducted to predict the percentage change scores in each domain using the T0 and T1 loading coefficients of components showing time effect with age, gender and cumulative antipsychotic dose as covariates.

**Results:**

Of the 30 spatial components of gray matter identified by SBM, loading coefficients of anterior cingulate cortex (ACC), anterior insula (AI) & inferior frontal gyrus (IFG), superior temporal gyrus (STG), middle temporal gyrus (MTG) and dorsal lateral prefrontal cortex (DLPFC) reduced with time in patients. The lower volume of AI & IFG at baseline and at 1 year related to poor improvement in positive and disorganization symptoms; lower volume of STG & MTG at baseline and 1 year predicted poor improvement in negative symptoms.

**Discussion:**

The baseline distribution of GM in AI & IFG, STG and MTG are predictive of the course of illness. The relationship between GM sources and symptom severity continues even after 1 year of naturalistic exposure to antipsychotic treatment. If judiciously combined with other available predictors of prognosis, source-based morphometric analysis can aid meaningful prognostication in schizophrenia.

